# Establishing Epidemic and Intensity Thresholds After a Major Change in Respiratory Virus Surveillance in Spain in 2020

**DOI:** 10.1111/irv.70136

**Published:** 2025-09-22

**Authors:** Ana María Puerto, José Eugenio Lozano, Marcos Lozano, Luca Basile, Gael Naveira‐Barbeito, Inés Guiu Cañete, Juan Antonio Linares Dópido, Ana Fernández Ibáñez, Ana Carmen Ibáñez Pérez, Daniel Castrillejo, Eva Rivas Wagner, María Ángel Valcárcel de la Iglesia, Esteban Pérez Morilla, Isabel Martínez‐Pino, Tomás Vega, Susana Monge, SiVIRA‐MEM collaborators

**Affiliations:** ^1^ National Centre of Epidemiology—Institute of Health Carlos III Madrid Spain; ^2^ General Public Health Directorate Regional Ministry of Health Valladolid Spain; ^3^ CIBER on Epidemiology and Public Health Madrid Spain; ^4^ Agència de Salut Pública de Catalunya, Generalitat de Catalunya Barcelona Spain; ^5^ Health Information Service General Directorate of Public Health Santiago de Compostela Galicia Spain; ^6^ Public Health Surveillance and Immunisations Service General Directorate of Public Health Zaragoza Aragón Spain; ^7^ Epidemiology Service General Directorate of Public Health, Extremadura Health Service Mérida Extremadura Spain; ^8^ Epidemiological Surveillance Service General Directorate of Public Health and Mental Health Oviedo Asturias Spain; ^9^ Epidemiology and Health Prevention Service General Directorate of Public Health La Rioja Spain; ^10^ Epidemiological Surveillance General Directorate of Public Health Melilla Spain; ^11^ Public Health Surveillance Unit General Directorate of Public Health, Canary Islands Health Service Las Palmas de Gran Canaria Spain; ^12^ Department of Epidemiology Regional Health Council Murcia Spain; ^13^ General Directorate of Public Health and Pharmaceutical Regulation Regional Ministry of Health and Consumption Seville Andalusia Spain; ^14^ CIBER on Infectious Diseases Madrid Spain

**Keywords:** acute respiratory infections, influenza, surveillance, thresholds, transmissibility

## Abstract

**Objectives:**

We estimated acute respiratory infections (ARIs) and influenza‐like illness (ILI) thresholds for SiVIRA, a new integrated surveillance system set up in Spain in 2020.

**Methods:**

We retrospectively extracted diagnostic codes from primary healthcare databases between Weeks 30/2010 and 39/2021, using methods similar to SiVIRA. Epidemic and intensity thresholds were estimated using the moving epidemic method (MEM).

**Results:**

The epidemic threshold for the 2024–2025 season was 400 cases per 100,000 persons for ARI and 36 for ILI, and medium, high, and very high intensity thresholds were 953, 1079, and 1139 for ARI and 167, 218, and 246 for ILI, estimating low intensity.

**Conclusions:**

Reconstructing the historical series using electronic health records was possible and allowed the estimation of thresholds. Experience gained will guide the choice of MEM parameters in the future.

Sentinel influenza surveillance had functioned in Spain since 1996 using the influenza‐like illness (ILI) European Commission (EC) case definition [1], relying on voluntary notification by sentinel doctors in primary healthcare (PHC). This system was disrupted in 2020 because of the COVID‐19 pandemic. Between 2020 and 2022, the European Centre for Disease Prevention and Control (ECDC) and the World Health Organization (WHO) published a series of guidelines to integrate influenza and SARS‐CoV‐2 surveillance [2, 3, 4]. SiVIRA, a new system for the integrated surveillance of acute respiratory infections (ARIs) was set up in Spain in 2021 [5].

One objective of SiVIRA is to assess the transmissibility of respiratory viruses each season by establishing epidemic and intensity thresholds [6]. However, the change in the surveillance system and the case definition after 2020 hinders the use of data predating COVID‐19 as historical data to estimate such thresholds. Our aim is to reconstruct historical ARI and ILI incidence data by using a system similar to SiVIRA on existing PHC databases since 2010, and estimate thresholds for the 2024–2025 season, using the 2023–2024 season for validation.

## 1 Description of SiVIRA

A comprehensive and integrated surveillance system to monitor a wide range of ARI in the post‐COVID‐19 era within SiVIRA has already been described [7]. SiVIRA has a first component of syndromic surveillance, followed by systematic laboratory testing and in‐depth data collection in a representative sample of syndromic cases. We focused on syndromic surveillance of ARI and ILI, two main indicators for transmissibility assessment [8].

Syndromic surveillance in SiVIRA moved from previous sentinel‐based and highly manual data collection of ILI to a more automated, sustainable, and resilient system based on electronic records and targeting the wider ARI case definition to include SARS‐CoV‐2 and RSV frequent clinical presentations. For identification of ARI cases, SiVIRA uses the EC case definition as reference [9]. However, case identification is implemented in practice through the extraction of ARI‐related diagnostic codes from episodes in PHC databases [5], with the subset related to influenza used to monitor ILI (Data S1).

## 2 Estimation of ARI and ILI Weekly Incidence From 2010 to 2024

**FIGURE 1:**
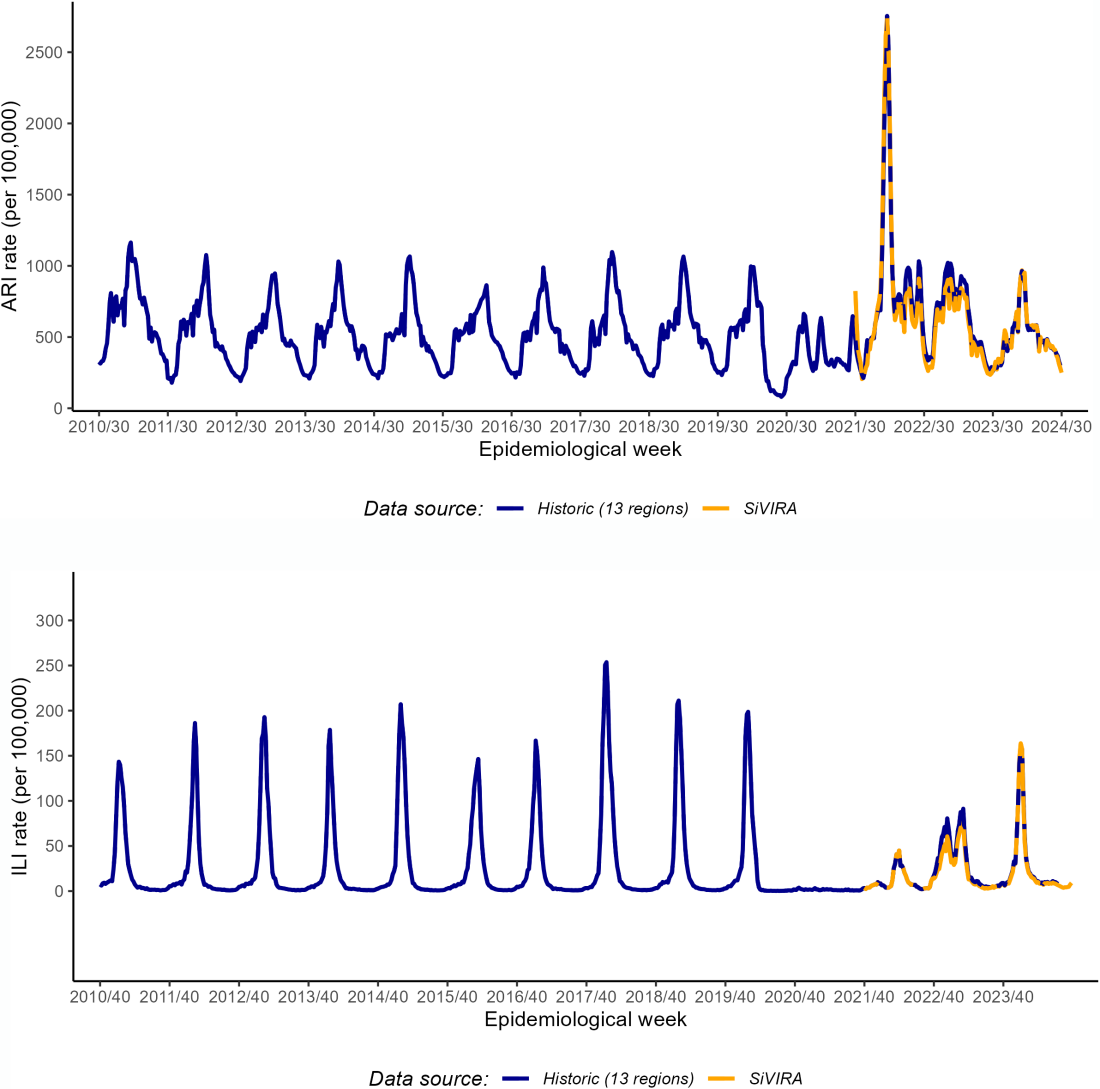
Acute respiratory infections (ARIs, upper panel) and influenza‐like illness (ILI, lower panel) weekly incidence reconstructed retrospectively from PHC records in 13 Spanish regions (seasons 2010–2011 to 2023–2024) or recorded in SiVIRA in up to 18 Spanish regions (seasons 2021–2022 to 2023–2024).

To reconstruct incidence series from Week 30/2010 up to Week 29/2024, the weekly number of episodes with ARI and ILI‐related diagnostic codes were retrospectively extracted from PHC databases in 13 of the 19 Spanish regions, using similar methods as the ones currently used in SiVIRA. These were divided by the corresponding population to derive incidence rates, and their weighted average was used to estimate national rates. Figure 1 shows a comparison between historical data and SiVIRA estimated rates. Overlapped series, between 2021 and 2024, show that the two estimates are highly comparable despite the different geographical coverage (with SiVIRA including up to 18 of 19 Spanish regions), suggesting low impact of existing geographical variability in the averaged national rates. We used the reconstructed series for seasons between 2010–2011 and 2020–2021 while, for seasons 2021–2022 onwards, we used incidence rates from SiVIRA.

ARI epidemics capture the circulation of a wide number of pathogens presenting with this common clinical picture and show a fairly stable shape and onset before the emergence of COVID‐19 (Figure 1a). However, after 2020, ARI incidence rates mainly reflect the dominant and unpredictable circulation of SARS‐CoV‐2, with multiple waves during the pandemic and with a particularly high peak in the 2021–2022 season, corresponding to the emergence of the Omicron SARS‐CoV‐2 variant. The sharp decrease at the beginning of 2020 likely reflects the severe disruption of the healthcare system during the early response to the pandemic and lack of coding at PHC. In contrast, circulation of influenza was absent or greatly reduced during the pandemic (Figure 1b). The 2023–2024 season was the first post‐pandemic season with restoration of pre‐pandemic characteristics for both ARI and ILI.

## 3 The Moving Epidemic Method (MEM)

The MEM is endorsed by WHO and ECDC for thresholds estimation [8, 10]. Detailed methodology and results have been previously published [11, 12]. Briefly, the MEM algorithm establishes the epidemic start and end of each season by maximizing the proportion of total incidence included within the minimum possible duration. The slope parameter is the threshold below which a percentage increment of cases classified within the epidemic when increasing the duration of the epidemic is considered negligible. An optimal choice of the slope parameter allows maximizing sensitivity (the model's ability to categorize epidemic weeks), specificity (ability to categorize non‐epidemic weeks) or a combined index, according to the validation analysis described by Vega et al. [10]. This classifies every weekly rate as pre‐epidemic, epidemic, or post‐epidemic.

The epidemic threshold is the 97.5th percentile of a normal distribution fitted to the *n* highest pre‐epidemic points each season. Intensity thresholds are estimated by fitting a log‐normal distribution to the *n* highest epidemic points from each season, with the 40th, 90th, and 97.5th percentiles corresponding to the medium, high, and very high thresholds. We used 30 points for both the pre‐epidemic and epidemic distributions, corresponding to *n* = 3 highest points per season, following the rationale by Vega et al. [10].

## 4 Implementing MEM in SiVIRA

**FIGURE 2:**
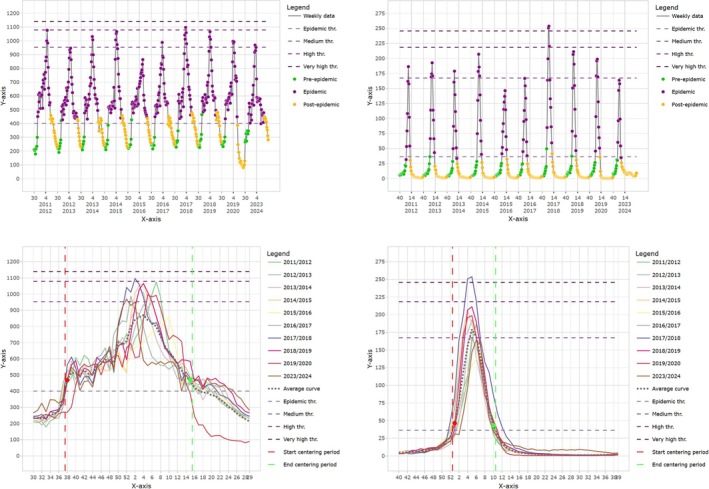
Epidemiological seasons used in the MEM model, with classification of weeks as pre‐epidemic, epidemic or post‐epidemic (upper panels) or overlapping, aligned to the first epidemic week each season (lower panels), for Acute Respiratory Infections (ARI, left panels) and Influenza‐like Illness (ILI, right panels). Y axis represent rates per 100,000 and X axis epidemiological weeks. Red and green dots represent the start and end of the average curve.

Out of available historical seasons, we removed 2020–2021, 2021–2022, and 2022–2023 because of unstable circulation of respiratory viruses due to the COVID‐19 pandemic and the response measures, using the last 10 remaining seasons to build the model (Figure 2). Seasons ran between Weeks 30 and 29 of the following year for ARI and between Weeks 40 and 39 for ILI. This captures the multiple non‐influenza viruses that normally increase with the start of the academic year in early September.

For ARI, we used a slope parameter of 1.7, resulting in an epidemic threshold of 400 cases per 100,000 persons, and 953 for medium, 1079 for high, and 1139 for very high intensity thresholds (Figure 2). The average epidemic started in Week 38 and lasted 30 weeks, capturing 74% of all ARI cases, with 85% sensitivity and 57% specificity in epidemic detection.

For ILI, we used a slope parameter of 2.6, resulting in an epidemic threshold of 36 cases per 100,000 persons, and 167 for medium, 218 for high, and 246 for very high intensity thresholds (Figure 2). The average epidemic started in Week 1 and lasted for 10 weeks, capturing 81% of all ILI cases, with 90% sensitivity and 98% specificity for epidemic detection.

The earlier start, longer duration, and low specificity of the ARI epidemic were expected, as ARIs reflect multiple pathogens giving a flatter and broader curve compared with ILI; thus, the slope parameter was optimized to enhance sensitivity. Influenza produces a more acute epidemic that is easier to detect with good sensitivity and specificity.

The intervals between intensity levels were narrow, possibly due to very homogeneous seasons, with low variability in the distribution of the three highest points per season. Though this entails low ability to discriminate across levels and reduces usefulness, thresholds were originally set so that most seasons might be considered normal (low or medium). Increasing the number of points taken per season might increase the width of intervals but could result in more false intensity alerts.

Finally, as a limitation, the validity of these thresholds could be affected by changes in coding systems and diagnostic practices over time as well as, for ILI, by the preferential use of influenza codes in laboratory‐confirmed cases.

## 5 Validation in the 2023–2024 Season

**FIGURE 3:**
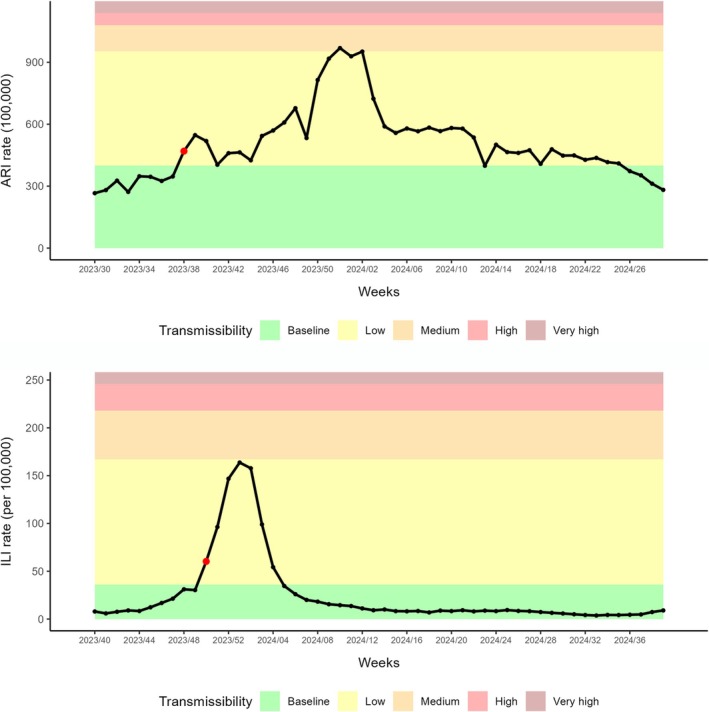
Estimated thresholds for the 2023–2024 season: acute respiratory infections (ARIs, upper panel) and influenza‐like illness (ILI, lower panel). The red dot indicates the first epidemic week.

We calculated thresholds for the 2023–2024 season using the method as described, but excluding the 2023–2024 season from the MEM model for this application, with thresholds remaining virtually unchanged. The epidemic spanned between Weeks 38/2023 and 13/2024 (28 weeks) for ARI and 50/2023 and 05/2024 (8 weeks) for ILI, reaching at the peak medium intensity for ARI and the upper limit of the low intensity level for ILI (Figure 3).

This resembles results of epidemic intensity for that season estimated within a more stable surveillance system in one Spanish region [7, 13] and validates the reconstructed national data for pre‐COVID seasons.

## 6 Prospective Use in the 2024–2025 Season

**FIGURE 4:**
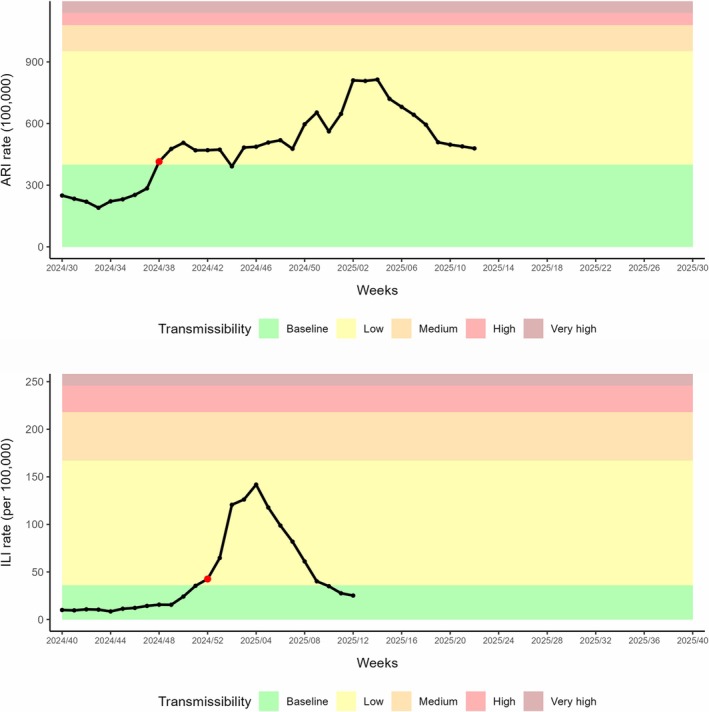
Estimated thresholds for the 2024–2025 season, up to Week 12/2025: acute respiratory infections (ARIs, upper panel) and influenza‐like illness (ILI, lower panel). The red dot indicates the first epidemic week.

As of Week 12/2025, estimated thresholds established the start of the ARI epidemic in Week 38/2024 and of the ILI epidemic in Week 52/2024, estimating low epidemic intensity for both, and with the ILI epidemic ending in Week 10/2025 (Figure 4) [14].

Experience during the current season will guide decisions on whether the slope parameter should be increased to enhance specificity for ARI, the number of points per season increased to widen intensity intervals, or alternatively, explore other available methods to calculate thresholds.

## 7 Conclusions

By reconstructing historical surveillance data using the extraction of diagnostic codes from episodes in PHC databases, we were able to overcome a critical change in the surveillance system. This allowed us to establish thresholds for ARI and ILI to prospectively inform prevention and control measures at the national level. Relevant geographical levels for decision‐making, including regions within Spain, can also greatly benefit from having locally established thresholds to also influence clinical practice and health planning. Finally, standardized estimation of transmissibility and intensity across countries within multinational structures such as ECDC and WHO can greatly contribute to the international respiratory virus risk assessment.

## Author Contributions

**Ana María Puerto:** methodology, data curation, formal analysis, writing – review and editing, visualization. **José Eugenio Lozano:** methodology, software, writing – review and editing, visualization, validation. **Marcos Lozano:** data curation, formal analysis, methodology. **Luca Basile:** validation, writing – review and editing, investigation, data curation. **Gael Naveira‐Barbeito:** investigation, validation, writing – review and editing, data curation. **Inés Guiu Cañete:** investigation, validation, writing – review and editing, data curation. **Juan Antonio Linares Dópido:** investigation, data curation, validation, writing – review and editing. **Ana Fernández Ibáñez:** investigation, validation, writing – review and editing, data curation. **Ana Carmen Ibáñez Pérez:** investigation, validation, writing – review and editing, data curation. **Daniel Castrillejo:** investigation, validation, writing – review and editing, data curation. **Eva Rivas Wagner:** investigation, data curation, validation, writing – review and editing. **María Ángel Valcárcel de la Iglesia:** investigation, data curation, validation, writing – review and editing. **Esteban Pérez Morilla:** investigation, data curation, validation, writing – review and editing. **Isabel Martínez‐Pino:** methodology, validation, writing – review and editing, investigation. **Tomás Vega:** conceptualization, methodology, supervision, writing – original draft, software. **Susana Monge:** conceptualization, methodology, supervision, writing – original draft.

## Ethics Statement

All data used for this study were collected as routine surveillance, and informed consent or official ethical approval was not required, as regulated by Royal Decree 2210/1995 of December 28 provided by the Ministry of Health and Consumer Affairs. Although individual informed consent was not required, all data were pseudo anonymised to protect patient privacy and confidentiality.

## Conflicts of Interest

The authors declare no conflicts of interest.

## Peer Review

The peer review history for this article is available at https://www.webofscience.com/api/gateway/wos/peer‐review/10.1111/irv.70136.

## Supporting information


**Data S1.** Supplementary table.

## Data Availability

The National Centre of Epidemiology has the mandate to collect, analyze, and disseminate surveillance data on infectious diseases in Spain. There is no direct access to the SiVIRA database, but data used for this study are available upon request to the corresponding author.
