# Supplementary material for: Establishing Epidemic and Intensity Thresholds After a Major Change in Respiratory Virus Surveillance in Spain in 2020
Source: Influenza Other Respir Viruses. 2025 Sep 22;19(9):e70136. doi: 10.1111/irv.70136 (PMC12453968; doi:10.1111/irv.70136)
Supplement: Supplementary file 1 — Data S1. Supplementary table. [file IRV-19-e70136-s001.docx]

**Establishing epidemic and intensity thresholds after a major change in respiratory virus surveillance in Spain after 2020**

**Supplementary Table**

**ICPC codes for Acute Respiratory Infection**

| **Categories** | **ICPC Codes** | **Title** | **Description** |
| --- | --- | --- | --- |
| **COVID-19** | A77 | Other viral diseases, unspecified |  |
|  | A77.01 | Coronavirus infection, unspecified |  |
|  | A77.04 (used regionally) | Suspected COVID-19 |  |
| **Acute Respiratory Infection** | R74 | Acute upper respiratory tract infection | Common cold, cephalea, coryza Acute infectious rhinitis  Acute rhinopharyngitis, nasopharyngitis  Acute pharyngitis Acute adenoiditis Acute laryngopharyngitis Upper respiratory infection  Acute upper respiratory tract infection |
|  | R76 | Acute tonsillitis | Other acute tonsillitis, Quinsy Other acute [pharyngotonsillitis](https://www.elsevier.es/en-revista-brazilian-journal-otorhinolaryngology-english-edition--497-articulo-pharyngotonsillitis-in-children-view-from-S1808869415308454) |
|  | R72 | Streptococcal pharyngitis/tonsillitis | Streptococcal pharyngitis/tonsillitis |
|  | R77 | Acute Laryngitis/tracheitis | Acute laryngitis Acute laryngitis/tracheitis Acute tracheitis Acute laryngotracheitis Laryngeal croup |
|  | R78 | Acute bronchitis/bronchiolitis, lower respiratory tract infection | Acute bronchiolitis Acute tracheobronchitis  Acute laryngotracheobronchitis Acute bronchitis Lower respiratory tract infection, unspecified acute  Acute lung infection, unspecified Bronchitis, unspecified |
|  | R78.00 | Bronchitis |  |
|  | R78.01 | Bronchiolitis |  |
| **Influenza** | R80 | Influenza | Influenza / influenza-like illness, Influenza Influenza confirmed by serology, Influenza |
| **Pneumonia** | R81 | Pneumonia | Bacterial/viral pneumonia, bronchopneumonia, influenzal pneumonia, Legionnaire's disease, pneumonitis |
|  | R81.01 | Bacterial pneumonia |  |
|  | R81.02 | Viral pneumonia |  |

**ICD-10 Codes for Acute Respiratory Infection**

| **ICD-10-CM Code range** | **Description** | **ICD-10-CM Codes included** |
| --- | --- | --- |
| **J00, J02 - J06** | Acute upper respiratory infections | J00 Acute nasopharyngitis [common cold] |
|  |  | J02 Acute pharyngitis Includes all descendant codes: J02.0, J02.8, J02.9 |
|  |  | J03 Acute tonsilitis  Includes all descendant codes: J03.0, J03.00, J03.01, J03.8, J03.80, J03.81, J03.9, J03.90, J03.91 |
|  |  | J04 Acute laryngitis and tracheitis Includes all descendant codes: J04.0, J04.1, J04.10, J04.11, J04.2, J04.3, J04.30, J04.31 |
|  |  | J05 Acute obstructive laryngitis [croup] and epiglottitis Includes all descendant codes: J05.0, J05.1, J05.10, J05.11 |
|  |  | J06 Acute upper respiratory infections of multiple and unspecified sites  Includes all descendant codes: J06.0 y J06.9 |
| **J09-J11** | Influenza | J09 Influenza due to certain identified influenza viruses Includes all descendant codes: J09.X, J09.X1, J09.X2, J09.X3, J09.X9 |
|  |  | J10  Influenza due to other identified influenza virus  Includes all descendant codes: J10.0, J10.00, J10.01, J10.08, J10.1, J10.2, J10.8, J10.81, J10.82, J10.83, J10.89 |
|  |  | J11 Influenza due to unidentified influenza virus  Includes all descendant codes: J11.0, J11.00, J11.08, J11.1, J11.2, J11.8, J11.81, J11.82, J11.83, J11.89 |
| **J12 - J18** | Pneumonia | J12 Viral pneumonia, not elsewhere classified Includes all descendant codes: J12.0, J12.1, J12.2, J12.3, J12.8, J12.81, J12.82, J12.89, J12.9 |
|  |  | J13 Pneumonia due to Streptococcus pneumoniae (this code does not have any descendant) |
|  |  | J14  Pneumonia due to Hemophilus influenzae (this code does not have any descendant) |
|  |  | J15 Bacterial pneumonia, not elsewhere classified  Includes all descendant codes: J15.0, J15.1, J15.2, J15.20, J15.21, J15.211, J15.212, J15.29, J15.3, J15.4, J15.5, J15.6, J15.7, J15.8, J15.9 |
|  |  | J16 Pneumonia due to other infectious organisms, not elsewhere classified Includes all descendant codes: J16.0, J16.8 |
|  |  | J17 Pneumonia in diseases classified elsewhere (this code does not have any descendant) |
|  |  | J18  Pneumonia, unspecified organism Includes all descendant codes: J18.0 , J18.1, J18.2, J18.8 y J18.9 |
| **J20 – J21** | Acute bronchitis and bronchiolitis | J20  Acute bronchitis  Includes all descendant codes: J20.0, J20.1, J20.2, J20.3, J20.4, J20.5, J20.6, J20.7, J20.8, J20.9 |
|  |  | J21 Acute bronchiolitis Includes all descendant codes: J21.0, J21.1, J21.8, J21.9 |
| **J22** | Unspecified acute lower respiratory infection | J22 Unspecified acute lower respiratory infection (this code does not have any descendant) |
| **U07.1** | COVID-19 | U07.1 COVID-19 |
| **B97** | Viral agents as the cause of diseases classified elsewhere | B97.0  Adenovirus as the cause of diseases classified elsewhere |
|  |  | B97.2 Coronavirus as the cause of diseases classified elsewhere  Includes all descendant codes: B97.21 y B97.29 |
|  |  | B97.4 Respiratory syncytial virus as the cause of diseases classified elsewhere |
|  |  | B97.81  Human metapneumovirus as the cause of diseases classified elsewhere |

**ICD-9 CM Codes for Acute Respiratory Infection**

| **ICD-9-CM Code range** | **Description** | **ICD-9-CM Codes included** |
| --- | --- | --- |
| **460, 462-465** | Acute upper respiratory infections | 460 Acute nasopharyngitis (common cold) (this code does not have any descendant) |
|  |  | 462 Acute pharyngitis (this code does not have any descendant) |
|  |  | 463 Acute tonsillitis (this code does not have any descendant) |
|  |  | 464 Acute laryngitis and tracheitis Includes all descendant codes: 464.0, 464.00, 464.01, 464.1, 464.10, 464.11, 464.2, 464.20, 464.21, 464.3, 464.30, 464.31, 464.4, 464.5, 464.50, 464.51 |
|  |  | 465 Acute upper respiratory infections of multiple or unspecified sites Includes all descendant codes: 465.0, 465.8, 465.9 |
| **466** | Acute bronchitis and bronchiolitis | 466  Acute bronchitis and bronchiolitis |
|  |  | 466.0 Acute bronchitis |
|  |  | 466.1 Acute bronchiolitis  Includes all descendant codes: 466.11, 466.19 |
| **480-486** | Pneumonia | 480 Viral pneumonia  Includes all descendant codes: 480.0, 480.1, 480.2, 480.3, 480.8, 480.9 |
|  |  | 481 Pneumococcal pneumonia (this code does not have any descendant) |
|  |  | 482 Other bacterial pneumonia  Includes all descendant codes: 482.0, 482.1, 482.2, 482.3, 482.30, 482.31, 482.32, 482.39, 482.4, 482.40, 482.41, 482.42, 482.49, 482.8, 482.81, 482.82, 482.83, 482.84, 482.89, 482.9 |
|  |  | 483 Pneumonia due to other specified organism Includes all descendant codes: 483.0, 483.1, 483.8 |
|  |  | 484.8 Pneumonia in infectious diseases classified elsewhere (this code does not have any descendant) |
|  |  | 485 Bronchopneumonia, organism unspecified (this code does not have any descendant) |
|  |  | 486 Pneumonia, organism unspecified (this code does not have any descendant) |
| **487** | Influenza | 487 Influenza  Includes all descendant codes: 487.0, 487.1, 487.8 |
|  |  | 488 Influenza due to identified avian influenza virus  Includes all descendant codes: 488.0, 488.01, 488.02, 488.09, 488.1, 488.11, 488.12, 488.19, 488.8, 488.81, 488.82, 488.89 |
| **079** | Viral and chlamydial infection in conditions classified elsewhere and of unspecified site | 079.0  Adenovirus infection |
|  |  | 079.3  Rhinovirus infection |
|  |  | 079.6 Respiratory syncytial virus (RSV) |
|  |  | 079.82 SARS-associated coronavirus |

**Reference**

National Epidemiological Surveillance Network (RENAVE). [Protocol for the surveillance of acute respiratory infections (ARI) in Primary Healthcare, Season 2024-25][Spanish]. Madrid, Instituto de Salud Carlos III; 4 December 2024. [Internet]. [cited 2025 Jan 7]. Available from: https://cne.isciii.es/documents/d/cne/protocolo-vigilancia-de-iras-en-espana-temporada-2024-25-1
